# Neurodevelopmental and functional outcome in hypoplastic left heart syndrome after Hybrid procedure as stage I

**DOI:** 10.3389/fped.2022.1099283

**Published:** 2023-01-16

**Authors:** Walter Knirsch, Alexandra De Silvestro, Michael von Rhein

**Affiliations:** ^1^ Pediatric Cardiology, Pediatric Heart Center, Department of Surgery, University Children's Hospital Zurich, Switzerland; ^2^ Children's Research Center, University Children's Hospital Zurich, Switzerland; ^3^ University of Zurich (UZH), Switzerland; ^4^ Child Development Center, University Children's Hospital Zurich, Switzerland

**Keywords:** congenital heart disease, outcome, HLHS, hypoplastic left heart syndrome, Hybrid

## Abstract

**Background:**

Patients with hypoplastic left heart syndrome (HLHS) undergoing staged palliation until Fontan procedure are at risk for impaired neurodevelopmental (ND) outcome. The Hybrid procedure with bilateral pulmonary artery banding, ductal stenting, and balloon atrioseptostomy may offer a less invasive stage I procedure compared to the Norwood stage I procedure avoiding early neonatal cardiopulmonary bypass (CPB) surgery. Despite altered fetal cerebral hemodynamics, the type of stage I procedure may be a covariate influencing ND outcome and functional outcome may also be altered due to postponing neonatal CPB surgery. Within this review, we analyzed ND outcome as well as functional outcome after Hybrid procedure as stage I procedure.

**Methods:**

The review analyzed original publications (OPs) published before March 15, 2022, identified by Cochrane, EMBASE, OVID, Scopus, and Web of science. An OP was included if short-to-long-term neurodevelopment outcome, brain development, somatic, and cardiac outcome in patients for HLHS and variants treated by Hybrid procedure were analyzed. In addition to database searches, we reviewed all references of the analyzed OP to obtain a comprehensive list of available studies. The author, year of publication, demographic characteristics of study population, study design (prospective or retrospective), study assessment, and main findings were summarized.

**Results:**

Twenty-one OPs were included with data of patients with ND outcome and functional cardiac outcome. Overall, there is an impaired mid-term ND outcome in patients with Hybrid procedure as stage I for HLHS. Only slight differences between stage I procedures (Hybrid vs. Norwood) in two comparing studies have been determined affecting right ventricular remodeling, short- and mid-term ND outcome, reduced brain growth until two years of age, sufficient quality of life, and altered hemodynamics influencing brain volumes and cerebral perfusion pattern.

**Conclusions:**

Despite some minor differences regarding the mid-term follow-up in patients with HLHS comparing Hybrid vs. Norwood procedure, its impact on ND outcome seems rather low. This may be explained by the large number of covariates as well as the small study populations and the different selection criteria for patients undergoing Hybrid or Norwood procedure as stage I.

## Introduction

Regarding congenital heart disease (CHD) as the most frequent birth defect with an incidence of 0.8% of life birth per year (1), the hypoplastic left heart syndrome (HLHS) is one of the most severe types of CHD accounting for 2%–3% of all CHDs or 2 of 10,000 live births per year (2, 3). Patients with HLHS undergoing staged palliation until Fontan procedure are at risk for impaired neurodevelopmental (ND) outcome (4). Impairment of brain development may start during *fetal life* in patients with HLHS due to an altered cerebral perfusion, impaired cerebral oxygenation, and reduced nutritional cerebral supply (5). *At birth*, this often results clinically in a microcephaly associated with a lower birth weight. The microcephaly resembles the clinical surrogate for a delay of brain growth and brain development, which has been determined as a delay of 3–4 weeks of gestational age at term (6, 7). The functional and cardiac outcome including body growth, pulmonary artery development, brain growth, hemodynamic findings, and myocardial function may be also affected in patients undergoing staged palliation for HLHS.

More than 95% of newborns with HLHS die after birth, if untreated. The urgent need for therapy for newborns with HLHS includes medical treatment with prostaglandin E1 for the patency of the ductus arteriosus and surgical palliation with perioperative pediatric ICU care due to early neonatal cardiopulmonary bypass (CPB) surgery. For over three decades, this surgical approach for HLHS started with early neonatal CPB surgery, the Norwood surgery as stage I procedure followed by hemi-Fontan or bidirectional cavopulmonary anastomosis during infancy as stage II (4–6 months), and completed by the Fontan procedure as stage III (2–3 years). However, overall survival of patients with HLHS is still limited with survival of 60%–65% at 5 years and 55% at 10 years, and the cardiac as well as extracardiac (ND outcome) morbidity remains a lifelong matter of concern (2, 3, 8).

In this context, postnatal surgical management appears to be a covariant factor influencing brain development as well as ND outcome (9). While the Norwood procedure is standard of care for the majority of patients with HLHS after birth, the perioperative invasiveness may influence ND outcome in up to 40% affected children including neuromotor ability, visual impairment, hearing loss, epilepsy, and cognitive and functional impairment (4).

As an alternative to Norwood stage I procedure, the Hybrid procedure including bilateral pulmonary artery banding, duct stenting, and balloon atrioseptostomy was developed in the early 1990s to improve outcomes among HLHS patients by providing a bridge to transplantation (10), in view of a still high mortality and morbidity. Pioneers of the Hybrid procedure started in 1993 (10, 11) with further use and modifications until today (12–14).

From the standpoint of impaired ND outcome, the Hybrid procedure was thought to be advantageous by postponing early neonatal CPB surgery to later potentially less invasive time periods in less immature and, therefore, less vulnerable brain structures avoiding overt brain damage (9).

Nowadays, the Hybrid procedure is frequently used in critically ill HLHS patients with associated risk factors such as prematurity, low birth weight, and comorbidity with the opportunity for bridging to Norwood procedure or heart transplantation, later on (15). This has been classified as Norwood alternative, salvage procedure, deferred Norwood procedure, pre-transplantation palliation, or univentricular–biventricular decision deferral (16).

The aim of this review was to provide an overview of the potential impact of Hybrid procedure on ND outcome compared to the Norwood procedure. The second aim was to analyze the functional outcome of the Hybrid procedure including somatic growth, pulmonary artery development, structural brain development, and myocardial function.

## Materials and methods

### Literature search

Systematic literature search using the terms “hypoplastic left heart syndrome” or “HLHS” and “neurodevelopment” and “hybrid” or “norwood” were performed on five electronic biomedical literature databases including Embase, Ovid, Web of science, Scopus, and Cochrane. During analysis, the reference list of each retrieved paper was further scanned for additional studies according to the selection criteria and publications analyzing functional cardiac outcome were added in the analysis. A second literature research was added by using the term (“Hypoplastic left heart syndrome” or “HLHS”) and hybrid and (neurodevelopmental* or “cerebral imaging” or “brain imaging” or “magnetic resonance imaging” or “MRI” or “ultrasound”).

### Selection criteria

Eligible studies for the review included mono- or multicentric original publications (OPs), with retrospective or prospective study design, analyzing short-to-long-term neurodevelopmental outcome after Hybrid procedure as initial stage I palliation in patients with HLHS as well as the functional cardiac outcome as defined above. Hybrid procedure was defined as combined bilateral pulmonary artery banding with ductal stenting or continuous prostaglandin E1 infusion to preserve ductal patency and balloon atrioseptostomy.

### Data extraction

Full OPs were extracted and checked critically for appraisal of evidence. Analyzed factors included name of the first author, year of publication, study design, number of patients, time, type and result of ND assessment, number of patients, type of CHD (HLHS/non-HLHS), type of stage I (Hybrid/Norwood), study assessment, and main findings.

## Results

**Figure 1:**
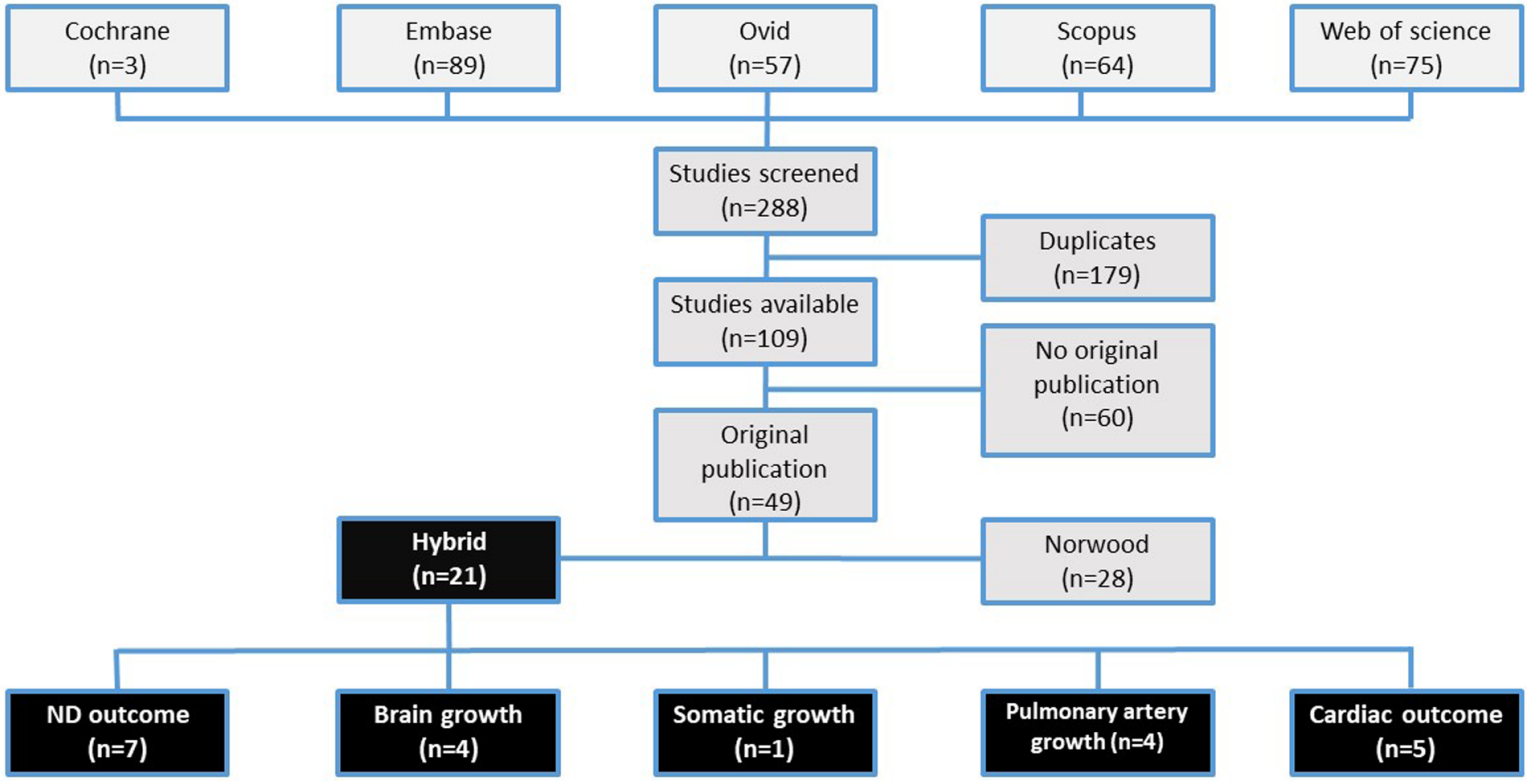
Search results.

The search was conducted on March 16, 2022, and resulted in 288 data literature records on Cochrane (*n* = 3), Embase (*n* = 89), Ovid (*n* = 57), Scopus (*n* = 64), and Web of science (*n* = 75) (Figure 1). After deduplication, a total of 109 publications remained. Sixty papers were further excluded due to their type as reviews (*n* = 23), conference papers (*n* = 23), case reports (*n* = 7), or others including book chapters, clinical trials, or editorials (*n* = 7). Search results are depicted in Figure 1.

Studies analyzing the ND outcome after Hybrid procedure (*n* = 7) as well as studies analyzing the outcome beyond ND including brain (*n* = 4), somatic (*n* = 1), and pulmonary artery growth (*n* = 4) and functional cardiac outcome (*n* = 5) are described in detail in Tables 1 and 2.

**Table 1:** Studies on neurodevelopmental outcome in patients with hypoplastic left heart syndrome with Hybrid procedure as stage I.

Study (Year)	Study design	Patients (*n*)	CHD type	Stage I	ND assessment	ND results
Cheatham et al. (17) (2015)	*Prospective single center*	18	HLHS	Hybrid	TIMP Bayley III	*2 months:* TIMP below average (−1 to −2 SD) *4 months:* TIMP below average (−0.5SD to −1SD) *6 months:* Bayley III (MCS) below norm
Khalid et al. (18) (2019)	*Retrospective single center*	24	HLHS (*n* = 9) Non-HLHS (*n* = 15)	Hybrid	Bayley III	*12 months:* abnormal (any delay) 11 of 24 (46%) LCS and MCS > 1 SD below norm (moderate delay) No difference between HLHS and non-HLHS
Knirsch et al. (19) (2012)^a^	*Prospective single center*	20	HLHS (*n* = 15) Non-HLHS (*n* = 5)	Hybrid (*n* = 9) Norwood (*n* = 11)	NMS Bayley II	*3 and 12 months:* NMS comparable in Hybrid and Norwood (increasing between 3 and 12 months) *12 months:* Bayley II (both MDI and PDI) in Hybrid and Norwood below norm, but comparable between both groups
Knirsch et al. (20) (2016)^b^	*Prospective single center*	16	HLHS Non-HLHS	Hybrid (*n* = 7) Norwood (*n* = 9)	WPPSI III Movement-ABC 2	*48 months:* total IQ and total motor score below norm
Reich et al. (21) (2017)	*Retrospective multicenter*	48	HLHS (*n* = 26) Non-HLHS (*n* = 22)	Hybrid (*n* = 25) Non-Hybrid (*n* = 27)	Neurological examination Bayley III TAPQOL	*2–3 years:* mild neurological abnormalities (35.4%). CCS and MCS comparable to norm. LCS below norm, but comparable for Hybrid and non-Hybrid, and HLHS and non-HLHS. QoL above norm
Reich et al. (22) (2019)^c^	*Retrospective multicenter*	25	HLHS (*n* = 23) HLHC (*n* = 2)	Hybrid (*n* = 21) Non-Hybrid (*n* = 4)	Neurological examination Bayley III	*2–3 years:* mild neurological abnormalities (23%). CCS, LCS, and MCS comparable to norm
Reich et al. (23) (2019)^d^	*Retrospective single center*	20	HLHS (*n* = 20)	Hybrid (*n* = 20)	Neurological examination Bayley III	*2–3 years:* mild neurological abnormalities (10%). CCS, LCS, and MCS comparable to norm

Bayley II/III, Bayley scales of infant and toddler development, 2nd and 3rd edition; CHD, congenital heart disease; CCS, cognitive composite score; HLHC, hypoplastic left heart complex; HLHS, hypoplastic left heart syndrome; LCS, language composite score; MCS, motor composite score; MDI, mental developmental index (MDI); ND, neurodevelopment; NMS, neuromotor score; QoL, health-related quality of life; TAPQOL, TNO-AZL preschool children quality of Life questionnaire; TIMP, test of impaired motor performance; WPPSI III, Wechsler primary preschool intelligence scale-III.

^a^ Neuromotor score was modified after Prechtl (24).

^b^ Follow-up study of four years of age of the former patient group of Knirsch et al. (19).

^c^ Part of the former patient group of Reich et al. (21), focusing on HLHS/HLHC patients and solitary treatment by Hybrid.

^d^ Part of the former patient group of Reich et al. (21), focusing on solitary diagnosis of HLHS and solitary treatment by Hybrid.

**Table 2:** Studies beyond neurodevelopmental outcome (cerebral, somatic, hemodynamic, and cardiac findings) in patients with hypoplastic left heart syndrome with Hybrid procedure as stage I.

Study (year)	Study design	Patients (*n*)	CHD type	Stage I	Study assessment	Main findings
Brain growth (cerebral imaging findings)						
Cheatham et al. (17) (2015)^a^	*Prospective single center case–control*	18	HLHS	Hybrid	TCD MCA	*2–6 months:* CBF lower in HLHS compared to controls. *6 months:* No correlations of CBF and Bayley III.
Heye et al. (25) (2017)^b^	*Retrospective multicenter case–control*	44	Single ventricle	Hybrid (*n* = 24) and Norwood (*n* = 6) Other (*n* = 14)	Cerebral MRI brain volumes	*2–3 years of age:* Total intracranial volume, total, deep gray, and white matter volumes reduced in single ventricle compared to controls. Absolute and (relative to intracranial) CSF volumes increased compared to controls, correlating with ND outcome.
Knirsch et al. (26) (2019)	*Retrospective multicenter case–control*	29	HLHS (*n* = 25) HLHC (*n* = 4)	Hybrid (*n* = 5) Norwood (*n* = 24)	Cerebral MRI brain volumes	*2–3 years of age:* Total brain volume, total and deep gray, white matter volumes reduced in HLHS/C compared to controls. Deep gray and white matter volumes more reduced after Norwood compared to Hybrid
Saiki et al. (27) (2013)	*Prospective single center*	Acute (*n* = 9) chronic (*n* = 26)	HLHS	Hybrid (acute *n* = 13) Hybrid (chronic *n* = 13) Norwood (*n* = 13)	Acute (0–7 days post Hybrid): Doppler flow MCA Chronic (before Stage II): OBI	*Acute:* Cerebral blood flow reduced after Hybrid *chronic:* OBI reduced after Hybrid > Norwood (impaired cerebral perfusion)
Somatic growth						
Chan et al. (28) (2017)^c^	*Retrospective single center*	62	HLHS	Hybrid (three-stage, *n* = 8; four-stage, *n* = 5) Norwood (*n* = 49)	Weight-for-age Height-for-age (Z-score)	*Body growth:* After Hybrid (Four stage > three stage^c^) more than after Norwood weight- and height-for-age declines until stage II and increases until stage III, Fontan procedure
Pulmonary artery growth						
Dave et al. (29) (2014)	*Retrospective single center*	48	HLHS Non-HLHS	Hybrid (*n* = 28) Norwood (*n* = 20)	Pulmonary artery indices (catheter)	*2–3 years of age:* Pulmonary artery indices smaller after Hybrid compared to Norwood before Fontan procedure.
Baba et al. (14) (2012)	*Retrospective single center*	75	HLHS Non-HLHS	Hybrid (*n* = 32) Norwood (*n* = 43)	Pulmonary artery indices (catheter)	*2–3 years of age:* Pulmonary artery indices (Nakata) smaller after Hybrid compared to Norwood before Fontan procedure. Higher PA reintervention rate.
Yerebakan et al. (30) (2016)	*Retrospective single center*	126	HLHS	Hybrid	Pulmonary artery indices (catheter)	*2–3 years of age:* McGoon ratio did not differ at comprehensive stage II and Fontan procedure.
Hemodynamic findings and myocardial function						
Cozzi et al. (31) (2017)	*Prospective single center*	13	HLHS	Hybrid	Mesenteric blood flow (Doppler)	Doppler mesenteric indices improve after Hybrid (coeliac artery > superior mesenteric artery)
Latus et al. (32) (2018)	*Retrospective multicenter*	86	HLHS	Hybrid (*n* = 44) Norwood (*n* = 42)	Myocardial function (MRI)	*2–3 years:* RVEDD larger, heart rate lower in Norwood compared to Hybrid. In both, preserved global RV function, but lower RV myocardial strain in Hybrid compared to Norwood. Pulmonary artery growth and rate of reintervention more was inferior using Hybrid.
Mah et al. (33) (2021)	*Retrospective multicenter*	46	HLHS	Hybrid (*n* = 20) Norwood (*n* = 26)	Myocardial function (Echo)	*Birth to 3 years:* RV size, RV FAC, calculated RV global radial shortening comparable after Hybrid and Norwood.
Kobayashi et al. (34) (2016)	*Retrospective single center*	23	HLHS	Hybrid (*n* = 23)	Myocardial function (Echo)	*Stage I to stage II:* MPI and ratio of systolic and diastolic durations were higher after Hybrid compared to controls.
Grotenhuis et al. (35) (2016)	*Retrospective single center*	76	HLHS	Hybrid (*n* = 34) Norwood (*n* = 42)	Myocardial function (Echo)	*Birth to 3 years:* RV FAC stage I to II smaller after Hybrid, after stage II comparable. After stage III RV size, RV size, and tricuspid regurgitation similar.

CBF, cerebral blood flow; CSF, cerebral spinal fluid; MCA, middle cerebral artery; MPI, myocardial performance index; MRI, magnetic resonance imaging; ND, neurodevelopment; OBI, oxygenation balance index between the lower and upper body; PA, pulmonary artery; RV, right ventricle; RVEDD, right ventricular end-diastolic diameter; RV FAC, right ventricular fractional area change; TCD, transcranial Doppler.

^a^ Study part of the study of Chetham et al. (17).

^b^ Study part of Knirsch et al. (27) (2017).

^c^ Three-stage pathway: Hybrid after birth -> Comprehensive stage II at 6 months -> Fontan procedure at 3 years of age; four-stage pathway: Hybrid after birth -> Norwood at 8 weeks of age -> stage II at 6 months -> Fontan procedure at 3 years of age.

A secondary search conducted on May 3, 2022, using (“Hypoplastic left heart syndrome” or “HLHS”) AND hybrid AND (neurodevelopmental* or “cerebral imaging” or “brain imaging” or “magnetic resonance imaging” or “MRI” or “ultrasound”) did not reveal further studies to be included.

### Neurodevelopmental outcome

The ND outcome in patients with HLHS undergoing Hybrid procedure has been determined in retrospective (*n* = 3), prospective (*n* = 4), monocentric (*n* = 3), or multicentric (*n* = 4) studies (Table 1). The number of analyzed patients per study ranged between 16 and 48, but the patient cohorts analyzed in the different studies were overlapping due to subsequent follow-up study with evaluation at an older age (4 years of age), or patient analysis focusing solitary on HLHS/hypoplastic left heart complex (HLHC) patients or solitary treatment by Hybrid and not comparing with Norwood (19, 21). Some studies additionally included infants with a “non-HLHS” diagnosis of CHD defined as HLHC with left heart hypoplasia according to small left heart structures due to dysbalanced atrioventricular septal defect with left ventricular hypoplasia (Table 1). The ND assessment was conducted at different time points starting at 2 months of age until 4 years of age. Most frequently, Bayley II or III scale was used as primary ND outcome parameter.

Overall, the results of ND assessment after Hybrid procedure as stage I were mildly below normal-referenced data of control subjects.

*At 2–4 months of age*, mild affection of motor development was found using the Test of Infant Motor Performance (TIMP) (37) with a mean TIMP score at 2 months of age of 63.9 ± 18.1 (−1 to −2 SD) and at 4 months of age of 108.3 ± 14.9 (−0.5 to −1 SD) (17).

*At the first half year of life (0–6 months)*, the neuromotor score [modified after (24) with a scaling of 0–18, i.e., 0 normal to 18 severely abnormal] was median (range) 4 (1–9) (19) after the Hybrid procedure.

*At 12 months of age*, the Bayley Scales of Infant Development II (Bayley II) were lower than the norm of 100 with median psychomotor development index (PDI) and mental development index (MDI) [PDI 57 (49–99), *P* < 0.001; MDI 91 (65–109), *P* = 0.002] (19). At the same age (12 months), Bayley III scores were comparable to the population norm for cognitive composite score (CCS) (95.2 ± 8.14) and 1 SD below the norm for language composite score (LCS) (93.6 ± 9.6) and motor composite score (MCS) (82.1 ± 11.9) (18).

*At 2–3 years of age,* despite mild neurological abnormalities, the median (range) LCS 97 (68–124) was below norm, while CCS with 100 (65–120) and MCS with 97 (55–124) were comparable to normative data (21) (Table 1).

*At 4 years of age,* cognitive outcome was determined by Wechsler Primary Preschool Intelligence Scale III (WPPSI III) and the Movement-ABC 2 for children after Hybrid were below the norm with median (range) IQ 88 (76–116) (20).

Comparative studies on ND outcome in children comparing Hybrid and Norwood procedure as stage I did not determine differences between cognitive, motor, or language development, neither using Bayley II scale at 1 year of age (19) nor using Bayley III scale at 2–3 years of age (21) or WPSSI III at 4 years of age (Table 1). Comparing the type of CHD (HLHS vs. non-HLHS) treated by Hybrid of ND outcome until preschool age ND outcome was similar (18, 19, 21).

### Cerebral imaging findings

Cerebral imaging findings were analyzed in four studies (Table 2) (17, 25–27). Transcranial Doppler cerebral blood flow (CBF) from the middle cerebral artery (17, 27) and cerebral MRI for assessment of brain volumes (26) showed lower CBF in infants (2–6 months of age) after Hybrid procedure as stage I compared to a control population (27), but showed no correlation with ND outcome at 6 months of age (17). Saiki et al. compared Doppler cerebral blood flow over a longer (chronic) time period between stage I and stage II with reduced oxygenation balance index between lower and upper body in patients after Hybrid procedure. While the total brain volume and total, deep gray, and white matter volumes were reduced in HLHS/C compared to controls at 2–3 years of age, the volume reduction of deep gray and white matter was more reduced after Norwood as stage I procedure compared to Hybrid procedure (26). Of note, intracranial cerebral spinal fluid volume was larger compared to healthy controls before stage III Fontan procedure and correlated with impaired ND outcome (25, 36).

### Somatic and pulmonary artery growth

Chan et al. compared in a small retrospective single-center study the weight-for-age and height-for-age (Z-score) somatic growth in three patient cohorts: Hybrid procedure with an early (within 4–6 weeks) switch to Norwood is defined as four-stage procedure (*n* = 5), Hybrid procedure with later comprehensive stage I/II is defined as a three-stage procedure (*n* = 8), and Norwood as stage I procedure (*n* = 49). The decline of weight-for-age (Z-score) between stage I and II was most distinct in the patient group of four-stage followed by three-stage procedure and the Norwood group (28), while in all three groups, there was a catch-up growth until stage III Fontan procedure (Table 2).

Limited pulmonary artery growth was described by smaller pulmonary artery indices after Hybrid procedure compared to Norwood patients before Fontan procedure at 2–3 years of age (29), while in another study, no differences of the pulmonary artery growth parameters such as McGoon ratio at comprehensive stage II and Fontan procedure in patients after Hybrid procedure were shown (30).

### Cardiac findings

Three studies analyzed the myocardial function comparing Hybrid and Norwood procedure using cardiac MRI or echocardiography (31–33). At 2–3 years of age, in cardiac MRI, right ventricle (RV) size was larger after Norwood compared to Hybrid procedure, while in both groups, global RV function was preserved but RV myocardial strain was reduced (32). This was also determined by echocardiography, with no differences until 3 years of age regarding RV size, RV function determined by right ventricular fraction area of change, or calculated global RV radial shortening after Hybrid and Norwood procedure (33) (Table 2).

## Discussion

The Hybrid procedure as stage I for the treatment of HLHS with PDA stenting, bilateral pulmonary artery banding combined with balloon atrioseptostomy, was developed as an alternative to the Norwood procedure in 1993 by Gibbs et al. (10), followed by the “Giessen group” in 1998 (11, 38). Further technical modifications of the Hybrid procedure were developed by combining PDA stenting by transpulmonary access immediately after pulmonary artery banding and delaying atrial septostomy to time of hospital discharge (Columbus and Sao Paolo) (12), and by including a reverse modified Blalock–Taussig shunt after pulmonary artery banding before PDA stenting (“Toronto group”) (14), these different technical modifications have been established in some centers for high-risk cases as a bridge to a delayed Norwood procedure (39). Despite less invasiveness of the Hybrid procedure in critical hemodynamic cases, the potential benefit of the Hybrid procedure remains the minimally invasive technique compared to the Norwood procedure by postponing early CPB surgery to a later time point with potential benefit on neurodevelopmental outcome (7). Although data on ND outcome after Hybrid procedure are limited due to small numbers of studies, this review focuses on ND and functional (cardiac) outcomes after Hybrid procedure.

In summary, this review on ND outcome of patients with HLHS after Hybrid procedure as stage I shows two major findings and consecutive challenges regarding this research topic for the future. First, the available data on ND outcome are limited concerning the number of patients being analyzed, comparing both treatment strategies as stage I (Hybrid vs. Norwood) in a prospective study protocol with a predefined neuroimaging follow-up using cerebral MRI with advanced cerebral MR perfusion techniques, volumetric assessment, metabolic, and microstructural (diffusion tensor imaging) assessment as well as a long-term ND outcome follow-up assessment. Second, the ND outcome follow-up assessment should be performed for a longer time period until adolescence and young adulthood and include more sophisticated test batteries for evaluation (executive function) of risk for alterations of brain maturation during development (40).

### Neurodevelopmental outcome

In the past, *ND outcome* of patients treated for HLHS has been analyzed by the single ventricle reconstruction trial of the Pediatric Heart Network Investigators (41). This network was started by enrolling a large population of HLHS patients from a number of centers in North America undergoing Norwood procedure with the primary focus comparing the type of aortopulmonary shunt procedure, i.e., the modified Blalock–Taussig shunt and right ventricular-to-pulmonary artery shunt (42). Nevertheless, the trial also assessed the ND outcome for HLHS after Norwood procedure at 14 months (43). In their study, the mean (SD) PDI (74 ± 19) and MDI (89 ± 18) scores were lower than normative means. Compared to the findings after Hybrid procedure at 1 year of age, PDI trended to be lower, while MDI was comparable after Hybrid procedure [PDI 57 (49–99), *P* < 0.001; MDI 91 (65–109), *P* = 0.002] (19). At 3 years of age, they found developmental delay in all domains of the Ages and Stages Questionnaire (ASQ) ranging between 17% and 35%, including communication, gross and fine motor, problem solving, and personal social interaction. Furthermore, behavioral attitudes, quality of life, and functional status were also reduced (44). Our review on ND outcome after Hybrid procedure as stage I for HLHS determines an impaired ND outcome in all domains of early childhood development until 3 years of age, comparable to the findings of the Pediatric Heart Network Investigators for the ND outcome after Norwood procedure as stage I (19–23, 26, 30, 31). This includes mild neurological abnormalities with rates between 10% and 35% (Table 1). A clear difference of ND outcome between Norwood and Hybrid procedure has not been determined, so far. Therefore, long-term ND follow-up studies comparing both procedures for stage I until age of adolescence or early adulthood assessing complete neuropsychological profiles with specific notice on cognitive ND outcome measurements such as memory, executive function, and processing speed are needed (45).

Two decades ago, ND outcome was compared between Norwood patients and primary heart transplantation at school age, showing for both groups an overall lower neurocognitive test results (full scale IQ 86 ± 14) without difference between groups stratified by surgical strategy (46). Nowadays, there are some studies determining lower to normal Bayley Scales III; differences between the Hybrid and Norwood patient groups were not evident as well as between the HLHS and non-HLHS patient groups (Table 1). Therefore, it is recommended that children with complex CHD are regularly monitored in a structured ND follow-up program to identify early developmental delay and introduce early interventions, when needed (47).

### (Interstage) mortality

According to the comparable results of ND outcome at 2–3 years of age after Hybrid and Norwood procedures, this has been also shown for overall *mortality* rate (including interstage mortality) comparing both treatment strategies. Lloyd et al. analyzed the preoperative condition and interstage mortality in Norwood and Hybrid procedures for HLHS (48). Although the patients after Hybrid procedure had a significantly higher Aristotle score, there was no difference for mortality at any stage. Yerebakan et al. reported single-center results of surgical mortality at stage I, comprehensive stage II, and Fontan procedure of 2.5%, 4.9%, and 0%, respectively, and an interstage mortality of 14.2% leading to a probability of survival of 77.8% at 10 years (30). Baba et al. compared survival after stage II and Fontan procedure after Hybrid and Norwood procedure as stage I, which were equivalent (14).

### Myocardial function

Differences in *myocardial function* in patients undergoing Hybrid and Norwood as stage I procedure have been analyzed (Table 2). While Kobayashi et al. focused on acute changes of myocardial function undergoing Hybrid procedure, they showed higher right ventricular myocardial performance index (MPI) in Hybrid patients before intervention, followed by a decrease of MPI after Hybrid procedure within 2 weeks (34). For the chronic changes of myocardial function, Grotenhuis et al. described similar indices of RV size over a longer follow-up for systolic and diastolic function throughout all stages of palliation for Norwood and for Hybrid procedure patients (35). In contrast, right ventricular remodeling seems to be altered when analyzing global radial shortening and deformation indices, as determined by the Toronto group (33). While right ventricular fractional area change remained normal until stage III Fontan procedure independent of Hybrid or Norwood procedure, the global radial shortening increases after stage I in both groups due to greater relative RV circumferential contraction and unchanged longitudinal strain until stage III. Nevertheless, the RV remodeling after Norwood and Hybrid procedure were comparable, and the impact of CPB bypass surgery during stage I was limited (33). Within the largest cohort comparative study between stage II and III, Latus et al. showed comparable preserved global RV pump function determined by cardiac MRI, but better RV strain, strain rate, and intraventricular synchrony after Norwood stage I, both factors not influencing all-cause mortality (32).

### Growth parameters

*Pulmonary artery growth* until stage III is more affected after Hybrid compared to Norwood procedure accompanied by a higher number of catheter interventions after Hybrid procedure (Table 2) (14, 18, 30, 32).

*Somatic growth* (determined as weight-for-age and height-for-age) declines after stage I. This decline continues until stage II and is most prominent for the critical ill neonates, who undergo Hybrid compared to Norwood procedure. Until stage III, Fontan procedure, a moderate increase of body growth follows (28). Nevertheless, after birth restricted body growth has been described for all growth parameters including body weight, body length, and head circumference, determined at birth before stage I, and is followed by a catch-up growth starting after stage II until stage III most prominent for the head circumference which is strongly correlated with the intracranial cerebral spinal fluid volume at 2–3 years of age (25).

On the other hand, *brain growth* determined for total brain volume and total, deep gray, and white matter volumes is reduced in a mixed population with HLHS and HLHC compared to healthy controls, while deep gray and white matter volumes are more reduced after Norwood stage I compared to Hybrid stage I (26).

Between stage I and stage II, *cerebral perfusion* determined by transcranial Doppler flow of middle cerebral artery that is either acute within the first week after Hybrid or until stage II is severely impaired after Hybrid (27). Similar findings were found in HLHS patients after Hybrid procedure as stage I with lower *cerebral blood flow* for 2–6 months compared to controls, but not correlating with ND outcome at 6 months of age (17). The oxygenation balance index between the lower and upper body was lower after Hybrid compared to Norwood procedure as stage I demonstrating impaired cerebral perfusion, which may be due to ongoing retrograde cerebral perfusion after Hybrid procedure as stage I (27). On the other hand, mesenteric perfusion determined by Doppler mesenteric indices improves after Hybrid procedure, which correlates with the potential beneficial effect for the prevention of necrotizing enterocolitis, which is a frequent complication in patients with HLHS by dysbalanced systemic (i.e., cerebral) and pulmonary perfusion (31).

### Limitations

No long-term data comparing ND outcome after Hybrid or Norwood procedure as stage I procedure are available so far, although the technique was developed more than two decades ago. The total number of patients studied remains low for risk factor analysis in a highly multifactorial setting.

## Conclusions

Overall, for comparing ND and functional outcome after Hybrid procedure, the numbers of studies are rather limited and differences between the two treatment options are rather small. Disadvantages of the Hybrid procedure as stage I include impaired pulmonary artery development, which may contribute to an impaired functional outcome after Fontan (49). Therefore, efforts are needed to enhance pulmonary artery growth as one of the most important prerequisite for later Fontan completion and long-term survival after Fontan completion until adulthood (50). This includes additional surgical procedures promoting symmetric pulmonary artery growth (51, 52) as well as optimizing catheter interventional procedure at different stages of treatment (53). So far, data available for children until preschool age show no clear advantage of Hybrid palliation on ND outcome, but long-term follow-up studies until adolescence or early adulthood are lacking, and the comparison of the two treatment strategies as stage I is still difficult due to a relevant selection bias. Nowadays, Hybrid stage I palliation for HLHS is used as a salvage procedure after birth obtaining hemodynamic stability (54) for high-risk HLHS patients with significant comorbidities (low birth weight or premature newborn or preterms), or is performed as a deferred Norwood procedure or a rapid two-stage Norwood procedure (55). Therefore, the Hybrid procedure for HLHS has become a kind of niche treatment option, and guidelines for the management of neonates and infants with HLHS summarize in most centers with an established Norwood program that Hybrid procedure is primarily used for high-risk neonates and to delay CPB bypass beyond the neonatal period (15).
